# From Donor Liver to Recipient Pulmonary Artery: Embolization of a Transected Central Venous Catheter During Liver Transplantation, A Rare Complication of Organ Procurement

**DOI:** 10.1002/ccr3.72962

**Published:** 2026-06-17

**Authors:** Fawaz Ahmed Prem Navaz, Laurence Weinberg, Nattaya Raykateeraroj, Thomas Moncrieff, Bernice Wang, Marie Sinclair, Helen Opdam, Manfred Spanger, Hin‐Boon Lew, Michael Fink

**Affiliations:** ^1^ Department of Anaesthesia Austin Health Melbourne Victoria Australia; ^2^ Department of Critical Care The University of Melbourne Melbourne Victoria Australia; ^3^ Department of Anesthesiology, Faculty of Medicine Siriraj Hospital Mahidol University Bangkok Thailand; ^4^ Department of Gastroenterology and Hepatology Austin Health Melbourne Victoria Australia; ^5^ Department of Intensive Care Austin Health Melbourne Victoria Australia; ^6^ Department of Radiology Eastern Health Melbourne Victoria Australia; ^7^ Department of Radiology Austin Health Melbourne Victoria Australia; ^8^ Department of Surgery The University of Melbourne, Austin Health Heidelberg Victoria Australia

**Keywords:** central venous catheter, embolization, liver transplantation, open disclosure, organ procurement

## Abstract

Central venous catheters (CVCs) are essential in transplantation but can rarely fracture and embolize, with donor‐to‐recipient transmission of catheter fragments scarcely described. A 62‐year‐old woman with hepatitis C–related cirrhosis and hepatocellular carcinoma underwent orthotopic liver transplantation from a brain‐dead donor with a left subclavian multi‐lumen CVC in situ at procurement. Postoperative chest radiography identified a new curvilinear opacity at the left hilum, and contrast‐enhanced computed tomography showed a 5 cm radiopaque structure in a segmental branch of the left lower lobe pulmonary artery, consistent with an embolized CVC tip. All recipient vascular access devices were removed intact, strongly suggesting a donor‐origin catheter fragment transplanted with the liver graft. Alternative explanations, including unrecognized recipient catheter fracture and pre‐existing foreign body, were considered but deemed less likely given the temporal relationship, imaging findings, and systematic device inspection. Percutaneous endovascular retrieval via femoral venous access was attempted but failed because of the fragment's distal location and probable endothelialization. Therapeutic anticoagulation was considered but not commenced because there was no radiological evidence of thrombus formation or pulmonary embolism, and substantial competing postoperative bleeding risk. The patient remained asymptomatic, and conservative management with clinical and imaging surveillance was adopted; at 6 months, she had excellent graft function and no cardiopulmonary sequelae. However, an uneventful short‐term follow‐up does not establish long‐term safety, and early retrieval remains the preferred strategy whenever technically feasible and clinically safe. This case represents, to our knowledge, a previously unreported mechanism of donor‐to‐recipient CVC fragment transmission in liver transplantation, with the most plausible explanation being unrecognized transection of the donor catheter during heart procurement with subsequent lodgment in hepatic venous outflow, although definitive proof is not possible in the absence of direct intraoperative recognition. It highlights an important systems‐level vulnerability at the interface between donor procurement, graft preparation, and recipient implantation. Modern multiorgan retrieval frequently involves multiple independent procedural teams, overlapping operative fields, and rapid transitions, creating fragmented responsibility for confirming removal and integrity of vascular access devices. This case underscores the importance of standardized vascular access reconciliation, careful inspection of donor venous structures, and early postoperative imaging. Structured vascular access reconciliation and verification protocols, analogous to surgical instrument counts and implant verification systems, may represent an important patient‐safety intervention in transplantation.

## Key Clinical Message

This case highlights a systems vulnerability in multiorgan procurement that risks inadvertent transfer of intravascular foreign bodies from donor to recipient. Prior to caval or atrial division, venous catheters should be removed and vessels inspected and palpated. Formal vascular access reconciliation and imaging review support timely detection and safe retrieval.

## 1 Introduction

Vascular access devices are indispensable in modern critical care and transplant medicine because they enable reliable hemodynamic monitoring, administration of vasoactive agents, and rapid transfusion during perioperative management. Despite their widespread use and general safety, central venous catheters (CVCs) are not without risk. Possible complications range from common mechanical and infectious events, such as arterial puncture, pneumothorax, catheter‐related bloodstream infection, or thrombosis, to uncommon but potentially serious mechanical failures [1]. The rarest complications include catheter fracture, transection, and subsequent embolization, which can result in migration of catheter fragments to the cardiac chambers or pulmonary vasculature.

Catheter embolization has been documented in a variety of settings, including insertion‐related trauma, excessive manipulation, or device fatigue during long‐term use [2, 3, 4]. However, reports of catheter fragment transfer between the organ donor and recipient are exceedingly uncommon [5, 6]. Such events pose unique diagnostic and management challenges, particularly in the context of organ transplantation, in which multiple teams and procedural stages are involved in graft procurement, preservation, and implantation.

Herein, we describe a unique and clinically significant case in which unrecognized transection of a CVC in a deceased donor most likely occurred during procurement and subsequently resulted in embolization of the catheter fragment into the recipient's pulmonary artery after reperfusion during liver transplantation. This case highlights a previously under‐recognized systems vulnerability within the interface between donor procurement, graft preparation, and recipient implantation, and underscores the need for meticulous, standardized inspection of vascular access devices during organ recovery and structured vascular access reconciliation protocols in order to prevent iatrogenic, device‐related complications during transplantation. Informed written consent was obtained from the patient for publication of this case report and accompanying images.

## 2 Case History

### 2.1 Deceased Donor Details

**FIGURE 1:**
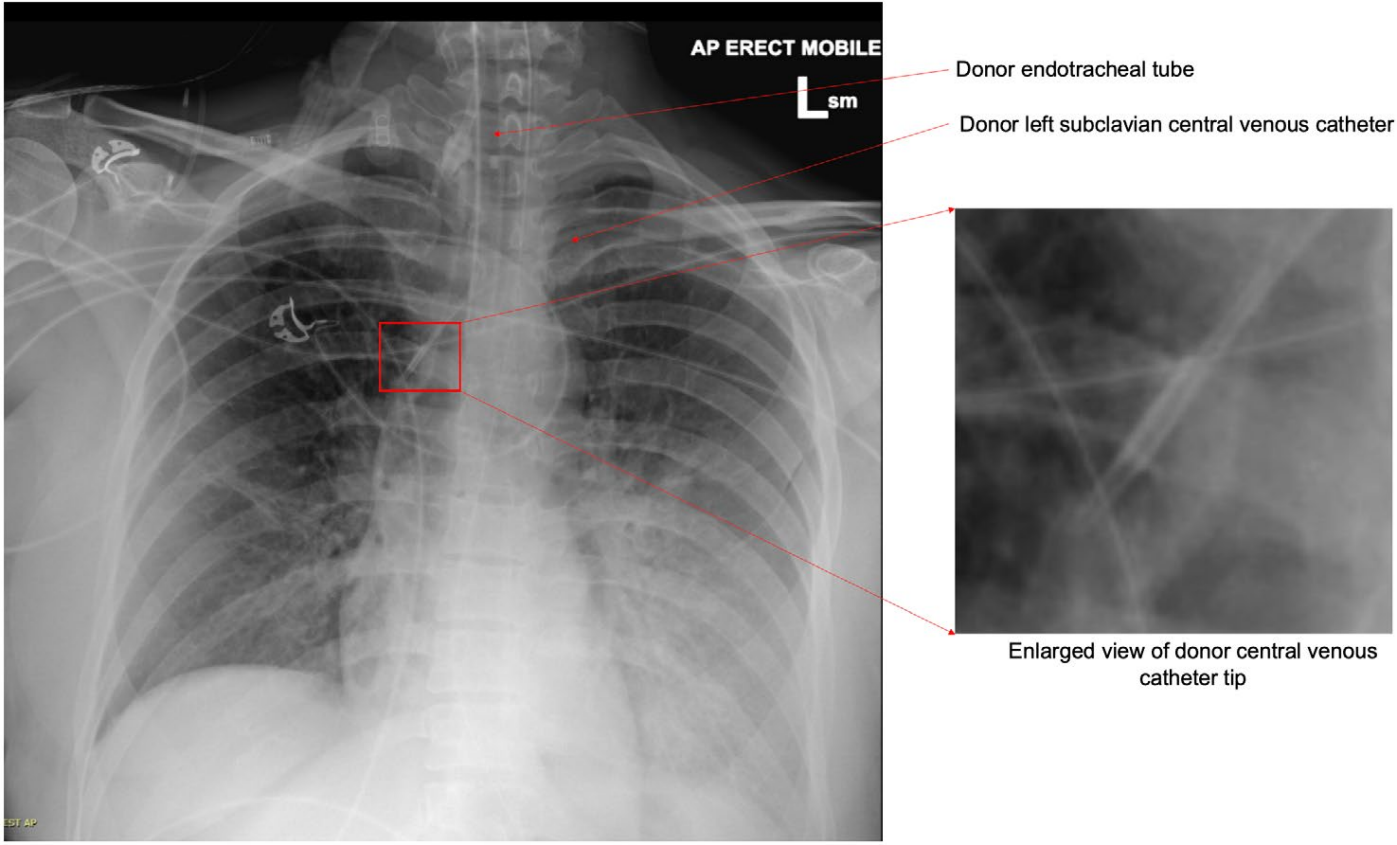
Chest radiograph of the donor before organ procurement with a left subclavian CVC in situ.

Organ procurement was performed via median sternotomy and midline laparotomy in a brain‐dead, hemodynamically stable, multi‐organ donor. A chest X‐ray immediately before procurement showed a multi‐lumen left subclavian CVC in situ (Figure 1). After systemic heparinization and cross‐clamping of the ascending aorta, cold cardioplegia and preservation solution were infused, and the heart–lung block was mobilized with circumferential dissection of the pericardium and pulmonary hila. The superior vena cava was divided above the cavo‐atrial junction; the inferior vena cava was transected at the diaphragmatic level to provide adequate caval cuffs; and the main pulmonary artery was divided just distal to the pulmonary valve annulus, to preserve a generous arterial cuff. The ascending aorta proximal to the brachiocephalic trunk was divided, while preserving adequate length for implantation, and the left atrium was opened posteriorly, with an incision extended around the confluence of the pulmonary veins to create a single left atrial cuff for the heart graft. After en bloc retrieval of the heart and lungs, the liver was procured through division of the infra‐diaphragmatic aorta above the celiac axis, and careful preservation of the common hepatic artery, portal vein, and suprahepatic and intrahepatic vena cava. The kidneys were recovered with intact renal arteries and veins, including Carrel aortic patches to facilitate subsequent arterial reconstruction. No catheter transection was recognized during procurement, and the donor catheter was not systematically withdrawn or definitively accounted for before caval transection.

### 2.2 Recipient Details

A 62‐year‐old woman (53 kg, 164 cm, body mass index 21.2 kg/m^2^) with hepatitis C–related cirrhosis (Model for End‐Stage Liver Disease score 12) and hepatocellular carcinoma was a suitable blood group and size match for the deceased‐donor liver graft. The recipient underwent a brain‐death, deceased‐donor orthotopic liver transplantation with duct‐to‐duct biliary reconstruction. After induction of general anesthesia, invasive monitoring was established with a left radial and right femoral arterial catheter, and standard central venous access, including a 9‐French sheath introducer and quad‐lumen CVC placed in the right internal jugular vein. A pulmonary artery catheter was advanced through the sheath introducer and positioned in the main pulmonary artery, and additional peripheral access consisted of a 14‐gauge intravenous cannula in the left arm and an 8.5‐French rapid‐infusion cannula in the right arm to facilitate high‐volume resuscitation and blood product administration.

The liver transplant proceeded without major intraoperative complications. Both the pre‐anhepatic (phase 1) and anhepatic (phase 2) phases were characterized by hemodynamic stability, low vasopressor requirements, acceptable lactate levels, and correction of mild coagulopathy with blood products. The anhepatic phase lasted 57 min, and graft reperfusion was uneventful apart from a modest, transient increase in vasopressor support at reperfusion, from which the patient was successfully weaned during the final (neohepatic) phase of the transplant. No intraluminal foreign body was identified during back‐table preparation of the donor liver. During the procedure, a total of 5 units of packed red blood cells and approximately 9 L of balanced crystalloid and albumin solutions were administered to maintain intravascular volume, optimize preload, and ensure adequate end‐organ perfusion.

## 3 Differential Diagnosis

**FIGURE 2:**
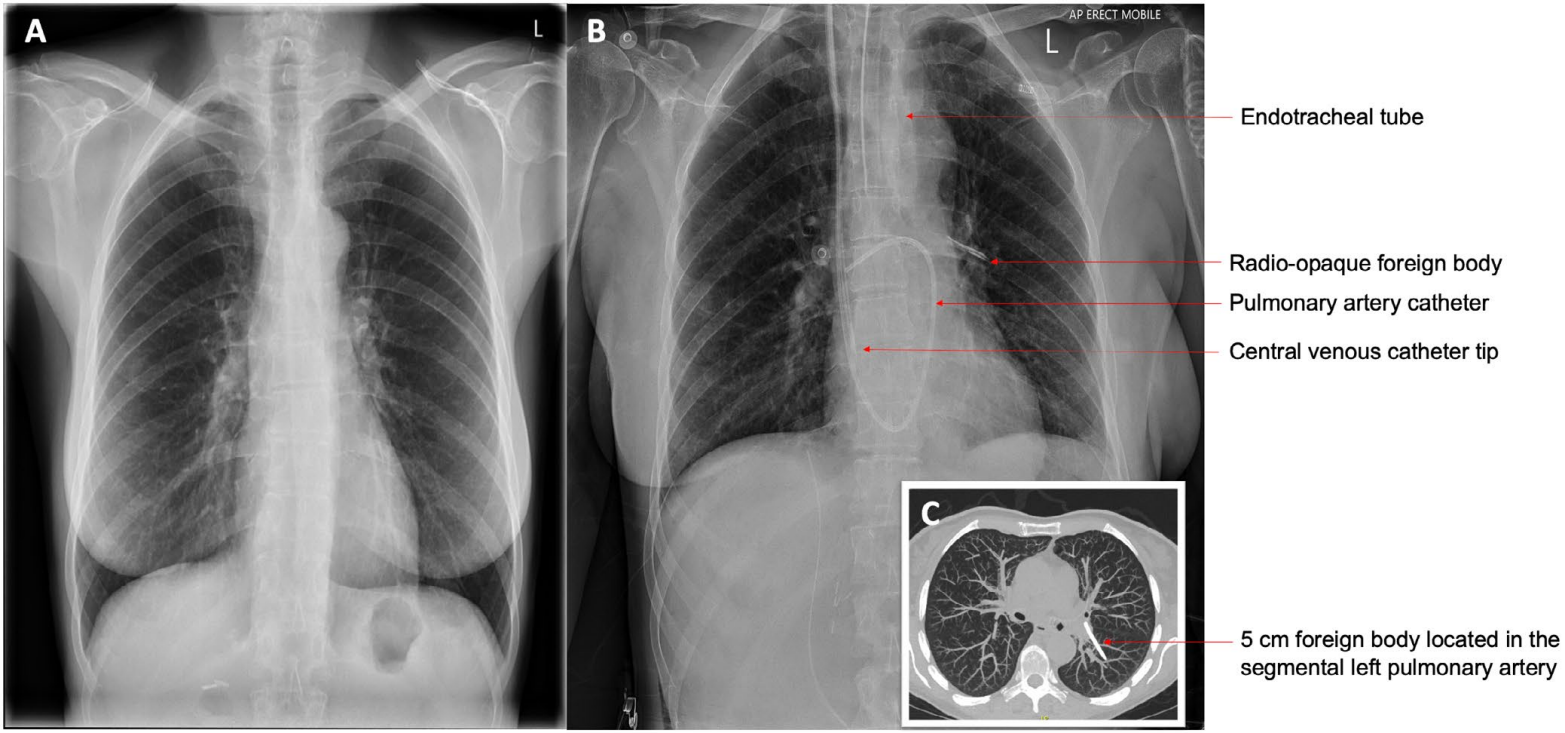
Preoperative (A) and postoperative (B) chest radiographs showing new linear opacity near the left hilar region, which was confirmed on chest computed tomography to be located within a segmental branch of the left lower lobe (C).

Postoperatively, a chest radiograph obtained at the time of intensive care unit admission demonstrated a new curvilinear opacity projected over the left hilar region that had not been present on preoperative imaging, thus prompting suspicion for an intravascular catheter fragment lodged within the left pulmonary arterial circulation. Subsequent contrast‐enhanced chest computed tomography confirmed a 5 cm linear radiopaque structure within a segmental branch of the left lower lobe pulmonary artery, whose appearance was consistent with an embolized CVC tip (Figure 2).

**FIGURE 3:**
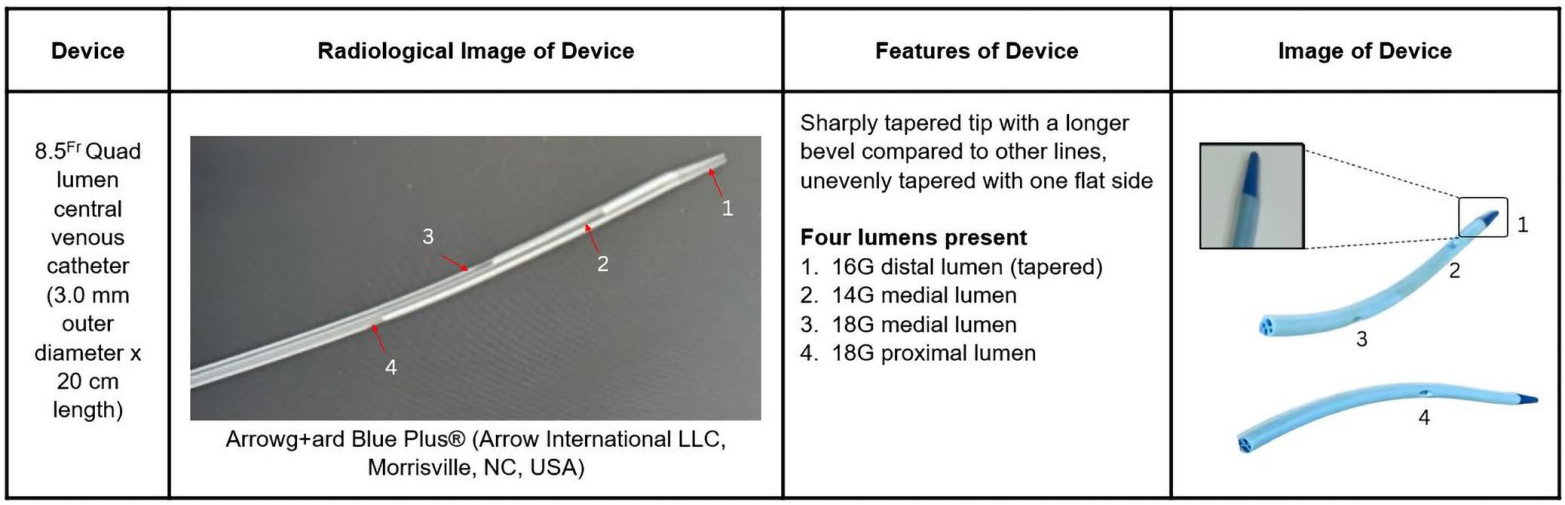
Radiographic appearance of CVC.

**FIGURE 4:**
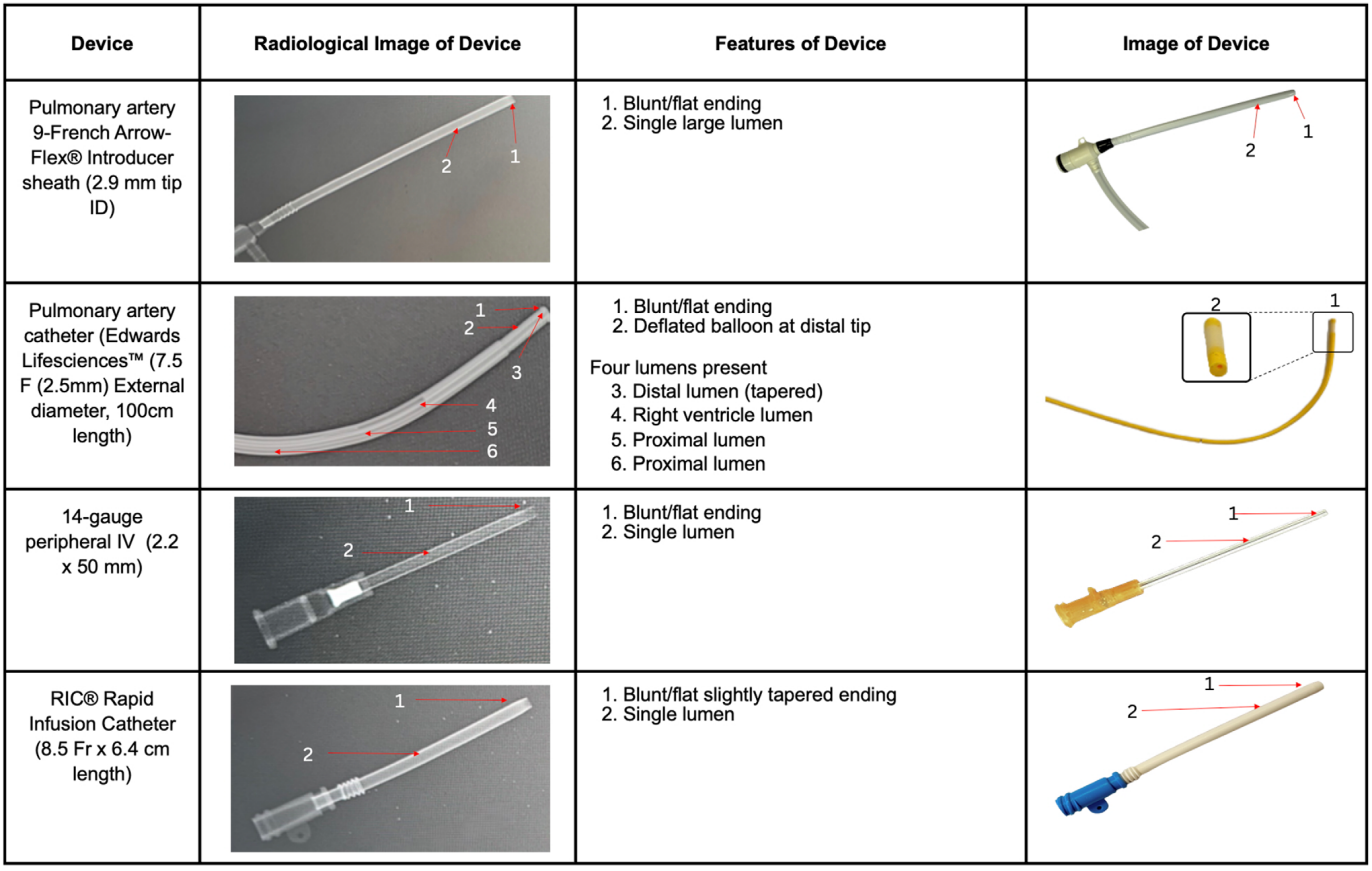
Radiographic appearance of vascular access devices.

Systematic evaluation of the CVC (Figure 3) and all other vascular access devices (Figure 4) used in the recipient, including removal, inspection, and radiographic comparison, indicated that each catheter had been retrieved intact, thus making an intraoperative recipient catheter fracture unlikely. Alternative explanations were considered, including: (1) unrecognized fracture of a recipient central venous catheter; (2) fracture of the pulmonary artery catheter or introducer sheath; (3) embolization of a previously retained intravascular foreign body unrelated to transplantation; and (4) imaging artifact. These alternatives were considered less likely because the opacity was absent on preoperative recipient imaging, computed tomography confirmed a true intravascular linear foreign body, and all recipient devices were inspected and radiographically correlated after removal, confirming intact distal tips and lumens. The size, configuration, and radiographic characteristics were highly suggestive of a catheter fragment of donor origin.

## 4 Treatment

**FIGURE 5:**
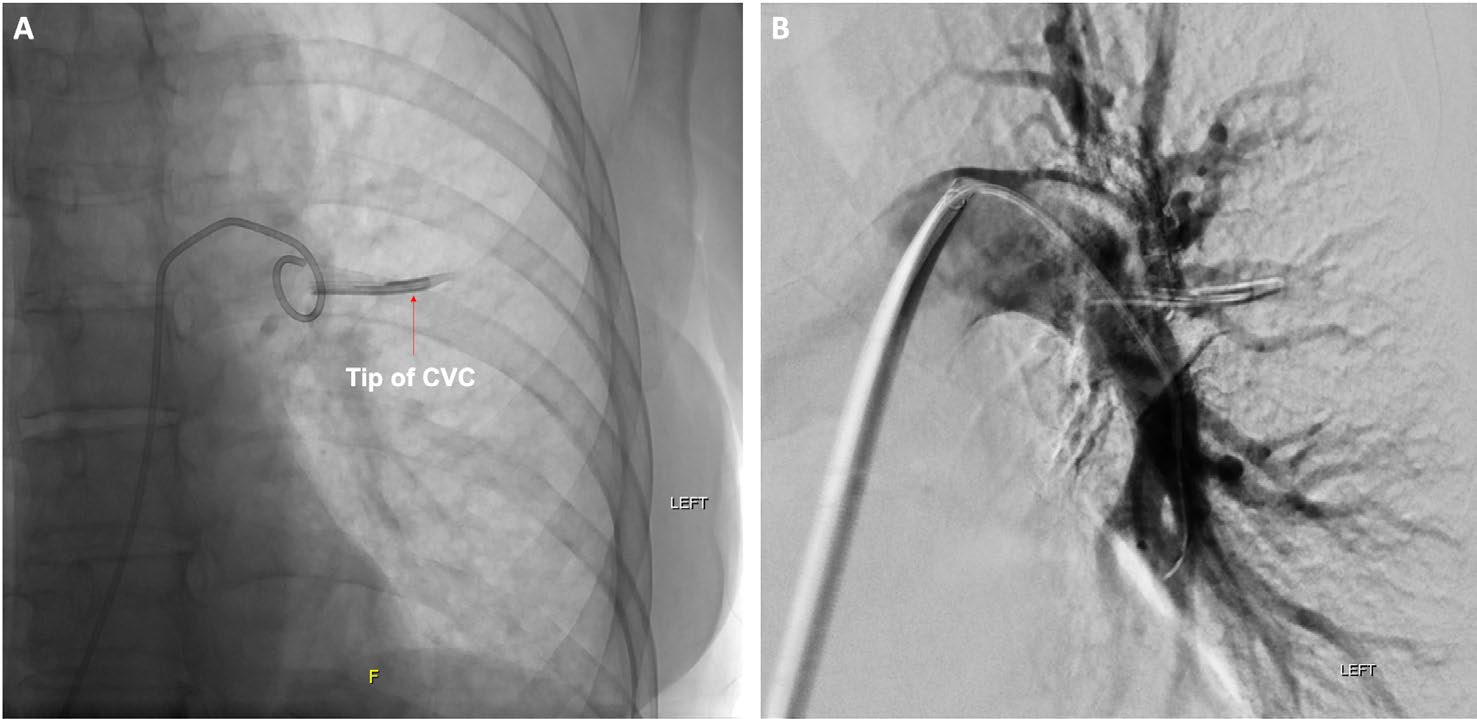
Unsubtracted image (A) and subtracted angiography (B) showing CVC tip in a segmental branch of the left pulmonary artery.

After the risks of endovascular retrieval of the intravascular catheter emboli were weighed against the risks of a retained foreign body, a decision was made to proceed with early image‐guided endovascular retrieval, recognizing the potential for long‐term thromboembolic, infectious, and mechanical complications associated with an intravascular catheter fragment. The literature generally supports early removal of intravascular foreign bodies whenever technically feasible, as progressive endothelialization and vessel wall incorporation may substantially increase technical difficulty and procedural risk associated with delayed retrieval. Under ultrasound guidance, an 8‐French sheath was inserted into the right common femoral vein, thus providing “uphill” venous access to the pulmonary circulation. Advancement of a guidewire and catheter through the right heart into the left main pulmonary artery enabled selective angiographic assessment of the suspected embolized fragment. A tubular foreign body was again visualized near the left hilar region and occluding a branch of the left pulmonary artery, in a position unchanged with respect to that in prior CT imaging (Figure 5).

Multiple retrieval attempts using different snaring devices targeted the exposed portion of the fragment within the segmental pulmonary artery. Despite adequate catheter positioning and repeated attempts, the foreign body could not be securely engaged and extracted, probably because of its distal location, orientation, and probable endothelialization or partial embedding within the vessel wall. Given the increasing procedural duration and escalating risk of vascular injury, further attempts were abandoned. Surgical retrieval was discussed during multidisciplinary review. However, the fragment was lodged distally within a segmental/subsegmental branch of the left pulmonary artery. Operative retrieval would likely have required thoracic surgical exposure with pulmonary arteriotomy, distal vascular exploration, or pulmonary parenchymal resection. Such intervention carried substantial risks including pulmonary arterial injury, major bleeding, postoperative respiratory compromise, infection, and prolonged recovery. The patient was recovering from recent orthotopic liver transplantation, remained clinically asymptomatic, had stable graft function, and had already undergone an unsuccessful but uncomplicated endovascular retrieval attempt. In this context, the multidisciplinary team considered the anticipated morbidity of thoracic surgical intervention disproportionate relative to the immediate clinical risk posed by the retained fragment. No immediate procedural complications occurred, including no evidence of access‐site bleeding, arrhythmia, hemodynamic instability, or acute deterioration in gas exchange. Given the failed retrieval, the foreign body was left in situ, and plans were made for ongoing clinical surveillance and interval imaging as part of the patient's post‐transplant follow‐up.

Therapeutic anticoagulation was considered during multidisciplinary discussion involving transplant surgery, hepatology, anesthesia, interventional radiology, and intensive care specialists. Ultimately, anticoagulation was not commenced solely for the retained fragment because there was no radiological evidence of thrombus formation, pulmonary infarction, right ventricular strain, or clinical pulmonary embolic syndrome. Importantly, the patient had recently undergone major liver transplantation and therefore had substantial competing postoperative bleeding risk. There are currently no evidence‐based guidelines specifically addressing anticoagulation for retained pulmonary arterial catheter fragments, particularly in transplant recipients. This decision reflected individualized multidisciplinary risk–benefit assessment rather than absence of concern regarding thrombotic risk.

Full open disclosure was provided to the patient and her family, including an explanation of the mechanism and cause of the retained intravascular foreign body, in accordance with contemporary open disclosure principles and statutory duty of candor requirements in Australia [7, 8]. This process included an apology, a factual description of the event, a discussion of potential short‐ and long‐term risks, and an outline of the planned management and measures instituted to prevent recurrence, in agreement with established guidance on communicating adverse events to patients.

## 5 Outcome and Follow Up

The remainder of her early postoperative course was notable only for hospital‐acquired pneumonia, which was successfully treated with intravenous antibiotics; serial Doppler ultrasound examinations indicated patent hepatic vasculature. The patient was discharged home after 9 postoperative days. At 6 months after liver transplantation, the patient remains asymptomatic, with normal physical examination findings and stable laboratory indices consistent with good liver graft function. Serial imaging and clinical reviews have demonstrated no evidence of arrhythmia, thrombosis, infection, or embolic phenomena attributable to the retained CVC fragment. No unplanned hospital presentations or interventions related to the retained fragment have occurred during this period, and the multidisciplinary team has continued a conservative management strategy with routine clinical and radiologic surveillance.

## 6 Discussion

This case illustrates an unusual mechanism of intravascular catheter embolization spanning organ procurement and implantation, with important implications for transplant practice and vascular access safety.

### 6.1 Proposed Mechanism and Rarity

**FIGURE 6:**
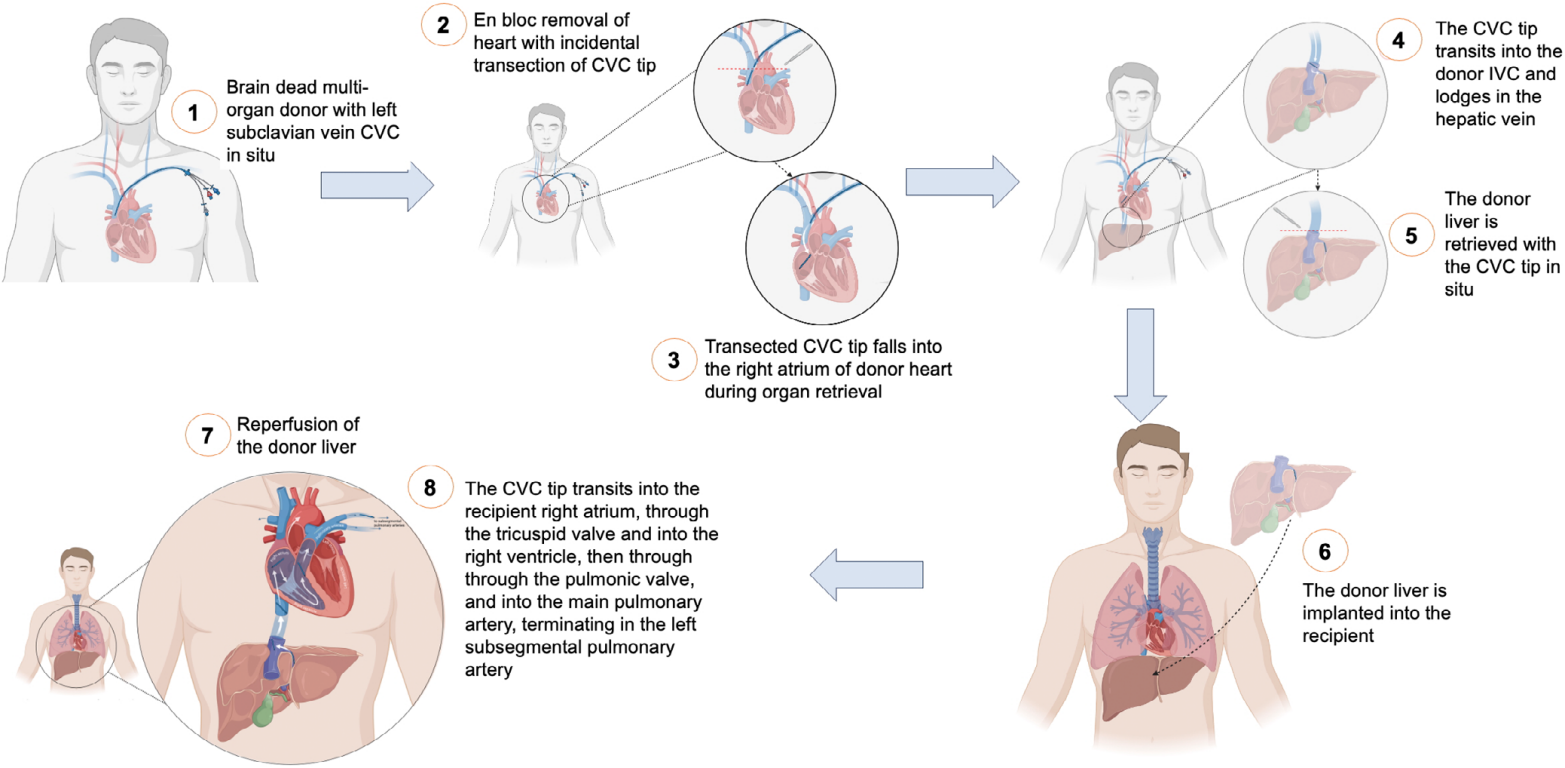
Postulated mechanism of embolization of the transected CVC.

Although the precise mechanism cannot be established with certainty in the absence of direct intraoperative recognition, the most plausible sequence is that, during donor heart procurement, the distal portion of the donor CVC was transected and dropped into the donor right atrium, then migrated into the inferior vena cava and lodged within a hepatic vein. During back‐table preparation of the donor liver, the inferior vena cava was inspected, but the intraluminal fragment was not recognized; consequently, the catheter tip remained within the hepatic venous outflow tract. After implantation and reperfusion of the graft in the recipient, the fragment probably migrated from the donor inferior vena cava into the recipient right atrium, then to the right ventricle, and through the pulmonary artery, and ultimately lodged in a left subsegmental pulmonary arterial branch (Figure 6). It is important to acknowledge that this proposed sequence remains inferential rather than directly observed. No catheter transection was recognized during procurement, no intraluminal foreign body was identified during back‐table preparation of the donor liver, and the residual donor catheter segment could not be retrospectively traced with certainty. Nevertheless, several findings strongly support a donor‐origin embolized catheter fragment: (1) pre‐procurement donor imaging demonstrated a left subclavian multi‐lumen CVC in situ; (2) postoperative recipient imaging demonstrated a new linear radiopaque intravascular structure not present on preoperative imaging; (3) all recipient vascular access devices were systematically removed and inspected intact; and (4) the morphology and radiographic appearance of the foreign body were highly consistent with a catheter fragment. We therefore present this mechanism as the most plausible explanation rather than a definitive conclusion. To our knowledge, this report describes the first reported case in which a donor CVC fragment was inadvertently transplanted within a liver graft and subsequently embolized into the recipient's pulmonary artery.

Intravascular catheter embolization, an extremely rare complication of vascular access in liver transplantation, has been estimated to occur in approximately 0.1%–0.3% of broader central venous device series [9, 10]. Therefore, this case adds to existing observations by demonstrating a donor‐to‐recipient transmission pathway and highlighting how a catheter fragment can evade detection despite standard intraoperative and back‐table checks.

### 6.2 Existing Literature on Catheter Fracture and Embolization

Beyond transplantation, most published data on catheter fracture and embolization consist of single case reports and small series [3, 4, 9, 10]. Larger reviews, such as those by Surov et al. [3] and others [11], have compiled several hundred cases of catheter embolization spanning multiple decades. These authors, along with Fisher et al. [12] have demonstrated that the pulmonary arterial circulation represents one of the most common final lodgment sites for embolized catheter fragments, together with the right atrium and right ventricle. This anatomical distribution is physiologically plausible because embolized venous foreign bodies traverse the right heart before lodging in progressively smaller pulmonary arterial branches. Those studies have indicated that totally implantable venous access devices and port systems account for most events, whereas percutaneous catheters represent a smaller fraction, and very few cases arise from perioperative CVCs in major surgery. Notably, the pulmonary artery has repeatedly been identified as one of the most frequent final lodgment sites, thus underscoring that asymptomatic pulmonary arterial fragments are not uncommon in this context. Additional series focusing on totally implantable CVCs have highlighted specific mechanisms such as “pinch off syndrome,” in which compression between the clavicle and first rib leads to progressive catheter damage and eventual intravascular fracture with embolization [11, 13].

### 6.3 Complications and Risk–Benefit Considerations

The clinical consequences of catheter embolization largely depend on the fragment's size, material, configuration, and final location. Reported complications include pulmonary artery embolization, arrhythmias, myocardial infarction, valvular or myocardial perforation with tamponade, cardiac arrest, and death [3, 14, 15, 16]. The fragment can act as a nidus for thrombus formation, thereby predisposing patients to pulmonary embolism as well as infectious complications, such as endocarditis, infected thrombus, pulmonary abscess, and systemic sepsis [3]. However, case reports have also documented patients in whom catheter fragments remained in situ for many years without apparent sequelae, thus prompting questions regarding the necessity and timing of removal in asymptomatic individuals [17, 18, 19]. However, it is important to recognize potential publication bias within the existing literature. The majority of available evidence derives from isolated case reports and small retrospective series, and cases with severe adverse outcomes may be underreported. Chronic retention carries ongoing risk of late complications including progressive endothelialization with increasing retrieval difficulty, thrombus formation, infective endocarditis, pulmonary abscess, septic embolization, arrhythmia, and vascular erosion. These observations suggest that chronic endothelialized fragments can remain clinically silent and that the risks of late retrieval may sometimes outweigh potential benefits.

### 6.4 Management in the Present Case

In the present case, the fragment lodged in a subsegmental branch of the left pulmonary artery in an otherwise clinically stable liver transplant recipient. A multidisciplinary team elected to attempt percutaneous retrieval, given concerns regarding potential long‐term thromboembolic or infectious complications in an immunosuppressed host. Despite optimal venous access, selective catheterization, and use of multiple snare techniques, the fragment could not be retrieved, probably because of its distal location, orientation, and partial embedding within the vessel wall. Given the risk of pulmonary artery injury or rupture with further manipulation, the procedure was terminated, and the fragment was left in situ.

It should be emphasized that early retrieval is generally preferred whenever technically feasible and clinically safe. Published experience suggests that progressive endothelialization, incorporation into the vessel wall, and local fibrosis may substantially increase technical difficulty and procedural risk associated with delayed retrieval. This decision aligns with the limited literature suggesting that endovascular retrieval is preferable when it is technically feasible and the risk is acceptable, whereas conservative management may be necessary when the fragment is peripherally lodged, the patient is asymptomatic, retrieval attempts have failed, and the likelihood of successful extraction is low or the procedural risk is judged to exceed anticipated benefit [20, 21, 22]. In this context, an individualized, patient‐centered assessment is essential, particularly in transplant recipients who face competing risks associated with immunosuppression, recent major surgery, and graft function. Key components of ongoing care include close clinical follow‐up, targeted imaging when indicated, and a low threshold for reassessing the retrieval strategy if symptoms or radiological changes arise [5, 23, 24].

In the transplant setting, unique considerations apply. Immunosuppressed recipients may be at elevated risk for infectious complications associated with retained intravascular foreign bodies. However, they also face competing risks related to recent major surgery, coagulopathy, and bleeding risk. In this case, anticoagulation was not commenced because there was no radiological evidence of thrombus and substantial bleeding risk in the immediate post‐transplant setting. The decision not to anticoagulate the patient reflected individualized risk–benefit assessment rather than absence of concern regarding thrombotic risk.

### 6.5 Learning Points and Preventive Strategies

This case extends beyond description of a rare technical complication. It highlights a previously under‐recognized systems vulnerability within the interface between donor procurement, graft preparation, and recipient implantation. In an era of increasingly complex multiorgan procurement pathways, structured vascular access reconciliation and verification processes may represent an important patient‐safety intervention analogous to surgical counts and implant reconciliation protocols used elsewhere in perioperative practice.

This extraordinary case highlights several pragmatic learning opportunities across the organ procurement and transplant continuum. Donor catheter management during procurement: During donor heart procurement, ensuring that all CVCs are fully withdrawn or at least retracted proximally before caval division or atrial transection is critical. Explicit verification of catheter position, withdrawal, and integrity should form part of a standardized procurement safety checklist or “time‐out”, with formal documentation of removal or secure positioning before cross‐clamp and explantation. Responsibility for this verification should be clearly assigned to a specific team member.

Back‐table graft inspection: During back‐table preparation of multiorgan grafts, particularly liver grafts with retained segments of donor inferior vena cava or hepatic veins, careful inspection and palpation of venous structures for intraluminal foreign bodies should be routine. If any doubt exists, intraoperative ultrasound or radiography of the graft may be considered to exclude occult devices. This inspection should be performed systematically and documented as part of graft preparation protocols.

Formal vascular access reconciliation: Transplant centers should implement clear protocols for reconciliation of all vascular access devices in both donors and recipients, analogous to surgical instrument counts and implant verification systems. This should include pre‐ and post‐procedure imaging review, device counts, systematic inspection of removed catheters to confirm intact tips and distal segments, and formal documentation and sign‐off that each catheter has been removed intact by both the procedural team and receiving team.

As an additional learning point, particular emphasis should be placed on meticulous removal of all vascular access devices after transplantation, including direct visual inspection of the tip and distal segment to confirm that the device is entirely intact before disposal or documentation. This aspect is particularly important when the donors had multiple invasive lines or prolonged intensive care stays.

Early postoperative imaging protocols: This case underscores the value of early postoperative imaging not only for graft assessment but also for detection of unexpected foreign bodies. Prompt recognition enables timely multidisciplinary discussion and intervention when retrieval is most likely to be successful, before significant endothelialization occurs. Together, our observations emphasize that, although intravascular catheter embolization in the liver transplant setting is highly rare, robust preventive systems and perioperative and postoperative vigilance are essential to decrease the likelihood of recurrence and to manage such complications safely when they do occur.

In conclusion, this case demonstrates a rare but clinically important mechanism of catheter embolization spanning organ procurement, implantation, and the early post‐transplant period. Although the precise mechanism cannot be established with certainty, the most plausible explanation involves unrecognized donor CVC transection that can result in intravascular foreign body transmission and subsequent pulmonary artery embolization in the recipient. Careful interpretation of imaging, systematic reconciliation of all vascular access devices, and a multidisciplinary, risk–benefit approach to retrieval enabled safe management of this complication while preserving excellent graft function. More broadly, the case demonstrates an important systems‐level vulnerability and underscores the need for structured vascular access reconciliation protocols, analogous to surgical instrument counts during procurement, meticulous inspection of donor venous structures, and rigorous verification of intact catheter removal in both donors and recipients, to prevent similar events in future liver transplants.

## Author Contributions

**Fawaz Ahmed Prem Navaz:** data curation, investigation, visualization, writing – original draft, writing – review and editing. **Laurence Weinberg:** conceptualization, data curation, formal analysis, investigation, methodology, project administration, supervision, validation, visualization, writing – original draft, writing – review and editing. **Nattaya Raykateeraroj:** data curation, investigation, resources, validation, visualization, writing – original draft, writing – review and editing. **Thomas Moncrieff:** resources, validation, visualization, writing – original draft. **Bernice Wang:** data curation, resources, validation, visualization, writing – review and editing. **Marie Sinclair:** conceptualization, data curation, investigation, methodology, project administration, validation, visualization, writing – review and editing. **Helen Opdam:** project administration, supervision, visualization, writing – review and editing. **Manfred Spanger:** data curation, investigation, methodology, resources, validation, visualization, writing – original draft, writing – review and editing. **Hin‐Boon Lew:** data curation, investigation, resources, validation, visualization, writing – original draft. **Michael Fink:** data curation, investigation, project administration, resources, supervision, validation, visualization, writing – original draft.

## Funding

The authors have nothing to report.

## Ethics Statement

The authors have nothing to report.

## Consent

Written informed consent was obtained from the patient for publication of this case report and accompanying images.

## Conflicts of Interest

The authors declare no conflicts of interest.

## Data Availability

Data sharing not applicable to this article as no datasets were generated or analysed during the current study.
